# A Millimeter-Wave 3D Imaging Algorithm for MIMO Synthetic Aperture Radar

**DOI:** 10.3390/s23135979

**Published:** 2023-06-27

**Authors:** Bo Lin, Chao Li, Yicai Ji, Xiaojun Liu, Guangyou Fang

**Affiliations:** 1 Aerospace Information Research Institute, Chinese Academy of Sciences, Beijing 100190, China; linbo20@mails.ucas.ac.cn (B.L.);; 2 Key Laboratory of Electromagnetic Radiation and Sensing Technology, Chinese Academy of Sciences, Beijing 100190, China; 3 School of Electronic, Electrical and Communication Engineering, University of Chinese Academy of Sciences, Beijing 100190, China

**Keywords:** 3D imaging, multiple-input-multiple-output synthetic aperture radar (MIMO-SAR), millimeter-wave (MMW), coherence factor

## Abstract

Multiple-input-multiple-output synthetic aperture radar (MIMO-SAR) is being studied and applied in more and more scenarios. However, there is still a certain distance away from real-time imaging using advanced algorithms. The traditional backpropagation algorithm (BPA) multi-accumulation integration is unsuitable for dealing with large-size scanning data, and the wavenumber domain algorithm requires the array to satisfy Nyquist sampling law in azimuth to avoid aliasing in imaging reconstruction. Based on these issues, a novel 3D imaging method is proposed for MIMO-SAR. An appropriate transformation and inverse Fourier transform (FT) is carried out for the frequency domain; thus, accumulation in the wavenumber domain is not required, which is easy to implement. The computational complexity of the algorithm is much lower than BPA and has better generalizability than the wavenumber domain algorithm. Coherence factor (CF) is also introduced to achieve sidelobe suppression. Proof-of-principle experiments were also carried out in the 92.5 GHz band based on the MIMO-SAR prototype system. Both simulation and experimental results of different distributed targets show good performance of imaging and do not lose the quality of image reconstruction.

## 1. Introduction

Millimeter-wave (MMW) and terahertz (THz) wave imaging systems with wide bandwidth have attracted more and more attention in various applications, including safety/security, non-destructive testing (NDT) [1,2,3,4,5], due to their millimeter/submillimeter-scale resolution and better penetration for nonmetal materials [3].

Millimeter-wave systems were typically designed based on a wideband transceiver combined with a 2D spatial scanning scheme in the early stage [6,7,8,9,10]. This results in show data collection and are not suitable for practical applications. For real-time security scenarios, lower system costs and higher data collection efficiency are required. To meet the requirements, a multiple-input-multiple-output (MIMO) array with a synthetic aperture in alone-track is an effective solution to achieve millimeter wave 3D imaging, which can acquire high-resolution images of concealed objects [11,12,13,14,15]. Compared with conventional synthetic aperture radar, MIMO-SAR has various advantages, such as a wide field of view, smaller system volume, and lower cost hardware system. Due to these advantages, MIMO-SAR is being studied and applied in more and more scenarios. 

It is easy to understand that the imaging algorithm is definitely one of the key techniques of the MIMO-SAR application. In array imaging applications, the most commonly used focusing algorithm is the backpropagation algorithm (BPA) [16]. Although BPA is accurate and reliable, its computation complexities increase linearly with the number of transceivers/samples and the number of desired image pixels. This means that a very high cost of computation time will be paid for 3D imaging reconstruction when using BPA, and this also means that BPA is not suitable for real-time imaging requirements. However, the greatest advantage of BPA is not limited to the array arrangement form, there is not strict requirements for array arrangement, which has a great advantage in hardware design. 

Another kind of focusing algorithm is the frequency-domain algorithm. This type of algorithm uses FFT, interpolation, and other operations to replace the complex iterative operations in BPA, which greatly improves image reconstruction efficiency. Zhuge et al. [17] first proposed a Range Migration algorithm (RMA, also known as the Omega-k algorithm) for MIMO 3D image reconstruction with a 2D array, which greatly improved algorithm efficiency compared with the BPA. Then, Zhu et al. applied RMA to MIMO-SAR based on the spherical wave decomposition. In the RMA algorithm, the interpolation operation can seriously affect the efficiency and quality of the image reconstruction, and a more accurate calculation also means a higher computational cost, which is inevitably different from the image reconstructed by BPA. Yang et al. proposed the phase shift migration (PSM) based on MIMO-SAR [18], which used accumulation instead of the interpolation operation in RMA. Although the accumulation operation will affect the imaging efficiency, the results will be more accurate. In addition, there are also image reconstruction algorithms based on frequency-wavenumber decoupling for MIMO-SAR [12,19]. The same reasonable phase transformation is used instead of interpolation to make the imaging results more accurate.

Since the frequency domain algorithms require FT and inverse FT operations in the azimuthal direction of the array, the placement of the array transceiver antennas needs to satisfy the Nyquist sampling law. The size of the transmitting and receiving antenna aperture, the size of the target area, and the distance between the array and the target all affect the spacing of the transmitting and receiving antenna units. To apply FT techniques in RMA and PSM conveniently, MIMO linear arrays should meet a uniform layout as much as possible, i.e., the element interval of the transmitting and receiving arrays is less than half-wavelength [18], which inevitably increases the cost. Therefore, sparse arrays are usually used to design the system, which causes scattering phenomena and disturbs the imaging quality.

In this paper, we proposed a fast imaging algorithm based on backpropagation (FBP) for 3D reconstruction under MIMO-SAR. The algorithm has the excellent characteristics of the BPA, which is possible to accurately reconstruct the target, but much faster than BPA. Meanwhile, the sampling interval in the azimuth is required to be looser, which can be applied to arbitrary sparse arrays; this is an advantage compared with frequency domain algorithms. Although its imaging speed is still somewhat different from the common frequency domain algorithm, it is applicable to arbitrary arrays and does not affect the reconstruction accuracy of the target. In addition, to suppress the sidelobe, CF is introduced to increase the power of the main flap. CF is combined with the proposed algorithm to achieve a rapid 3D reconstruction of the objective function with a low sidelobe. Finally, a MIMO-SAR prototype system was designed for proof-of-principle experiments in the 92.5 GHz band. Both simulation and comparison experiments show the effectiveness of the proposed algorithm.

The rest of this paper is organized as follows. Section 2 first presents the formulation and implementation of the modified algorithm for 3D reconstruction under a MIMO-SAR illuminating geometry; it also presents the computational complexity and error of the proposed algorithm. Simulations and experiments for verifying the algorithm are presented in Section 3. Finally, the discussion and conclusions are shown in Section 4 and Section 5.

## 2. Formulation

### 2.1. Traditional BP Algorithm for MIMO-SAR Imaging

**Figure 1:**
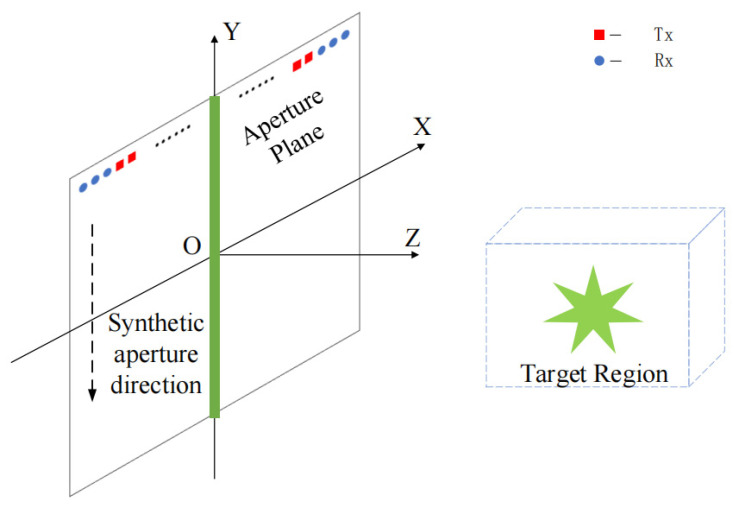
Imaging geometry based MIMO-SAR.

Consider the MIMO-SAR imaging geometry given in Figure 1. A linear MIMO array that is parallel to the *X*-axis scans along the height direction *Y*, which is located at a plane z=0 m to illuminate a target in front of the aperture. The coordinates of the transmit and receive antennas are defined as $\left(x_{t},y_{l},0\right)$ and $\left(x_{r},y_{l},0\right)$ while the location of the scattering point is represented by $\left(x,y,z\right)$. To obtain a certain resolution in the z direction for 3D imaging, the transmitted wave should have a certain bandwidth. Hence, we choose a step-frequency continuous wave (SFCW) signal as the transmitted waveform. Under this MIMO-SAR imaging scheme, the corresponding received signal [20] can be denoted as:

(1) $$\begin{array}{c} sS\left(x_{t},x_{r},y_{l},k_{i}\right)={∭}_{V}dxdydz\cdot o\left(x,y,z\right)\exp\left[-jk_{i}\left(R_{T}+R_{R}\right)\right]\\ \;\;\;={∭}_{V}dxdydz\cdot o\left(x,y,z\right)\exp\left(-jk_{i}R_{M}\right) \end{array}$$

where $o\left(x,y,z\right)$ denotes the target reflectivity function, $k_{i}=2\pi f_{i}/c$$\left(i=1,2,\ldots ,N_{k}\right)$ is the wavenumber, $N_{f}$ is the number of wavenumber domain, respectively, and

(2) $$R_{T}=\sqrt{{\left(x_{t}-x\right)}^{2}+{\left(y_{l}-y\right)}^{2}+z^{2}}$$

(3) $$R_{R}=\sqrt{{\left(x_{r}-x\right)}^{2}+{\left(y_{l}-y\right)}^{2}+z^{2}}$$

(4) $$R_{M}=R_{T}+R_{R}$$

where $R_{T}$ and $R_{R}$ are the distance between the target and the transmit and receive elements, and $R_{M}$ is the distance from the round trip.

Assume that $k_{i}$ is sampled uniformly, such as

(5) $$k_{i}=k_{0}+\left(i-1\right)\Delta k$$

where $k_{0}$ is the starting wavenumber, and Δk is the sample interval.

Then, the traditional BP imaging algorithm for MIMO-SAR systems can be easily expressed as

(6) $$\begin{array}{l} I_{BP}\left(x,y,z\right)\\ =∭\int dx_{t}dx_{r}dy_{l}dk_{i}\cdot sS\left(x_{t},x_{r},y_{l},k_{i}\right)\exp\left[jk_{i}\left(R_{T}+R_{R}\right)\right] \end{array}$$

For each spatial grid point in the region of interest (ROI), a complex quadruple integration is necessarily performed to obtain the reflectance of the point. Thus it is necessary to optimize the traditional BP algorithm.

### 2.2. The Proposed Algorithm

Through the analysis of the traditional BP algorithm in the previous section, it can be found that the time-consuming operation is a quadruple integration, so the integration step is considered reduced. Meanwhile, to ensure the applicability of the algorithm to arbitrary arrays, a certain transformation is taken to eliminate this integration layer for the wavenumber $k_{i}$ in this section.

Substituting (5) to (1), we have

(7) $$\begin{array}{c} sS\left(x_{t},x_{r},y_{l},k_{i}\right)={∭}_{V}dxdydz\cdot o\left(x,y,z\right)\exp\left(-jk_{0}R_{M}\right)\\ \cdot \exp\left[-j\left(i-1\right)\Delta kR_{M}\right] \end{array}$$

Introduce a Fourier transform pair with respect to $k_{i}$:

(8) $$\begin{array}{ll} R_{n} & =\left(n-1\right)\Delta R\\ & =\left(n-1\right)\frac{2\pi }{\Delta k}=\left(n-1\right)\frac{2\pi }{B_{k}}\left(N_{f}-1\right) \end{array}$$

where $B_{k}=\left(N_{f}-1\right)\Delta k$ is the wavenumber domain bandwidth. Substitute $R_{n}$ for $R_{M}$, Equation (4) can be written as:

(9) $$\begin{array}{ll} sS\left(x_{t},x_{r},y_{l},k_{i}\right) & ={∭}_{V}dxdydz\cdot o\left(x,y,z\right)\exp\left(-jk_{0}R_{M}\right)\\ & \cdot \exp\left[-j\left(i-1\right)\Delta kR_{n}\right]\cdot E\left(x_{t},x_{r},y_{l},k_{i}\right)\\ & \approx {∭}_{V}dxdydz\cdot o\left(x,y,z\right)\exp\left(-jk_{0}R_{M}\right)\\ & \cdot \exp\left[-j\left(i-1\right)\Delta k\left(n-1\right)\Delta R\right] \end{array}$$

(10) $$E\left(x_{t},x_{r},y_{l},k_{i}\right)=\exp\left[-j\left(i-1\right)\Delta k\left(R_{M}-R_{n}\right)\right]$$

where $E\left(x_{t},x_{r},y_{l},k_{i}\right)$ in Equation (10) is the phase error obtained from $R_{n}$ approximating $R_{M}$, small ΔR can reduce the phase error.

Then, $ss\left(x_{t},x_{r},y_{l},n_{k}\right)$ can be obtained simply by carrying a 1D inverse Fourier transform (FT) for $sS\left(x_{t},x_{r},y_{l},k_{i}\right)$:

(11) $$\begin{array}{ll} ss\left(x_{t},x_{r},y_{l},n_{k}\right) & =IFFT_{k_{i}}\left[sS\left(x_{t},x_{r},y_{l},k_{i}\right)\right]\\ & ={∭}_{V}dxdydz\cdot o\left(x,y,z\right)\exp\left(-jk_{0}R_{M}\right)\delta \left(n_{k}\right) \end{array}$$

(12) $$n_{k}=\mathrm{int}\left(\frac{R_{M}}{\Delta R}\right)$$

where $\mathrm{int}\left(\cdot \right)$ is the round-off operation, and the function $\delta \left(n_{k}\right)$ is defined as:

(13) $$\delta \left(n_{k}\right)=\left\{\begin{array}{c} 1\;,\;n=n_{k}\\ 0\;,\;n\neq n_{k} \end{array}\right.$$

In Equation (9), we have the approximation $R_{n}\approx R_{M}$. Minimize the sampling interval ΔR to reduce the phase error; that is, the number of inverse FT points should be enough. Assume $MN_{k}$ is the number of inverse FT output, Equation (11) can be rewritten as:

(14) $$ss\left(x_{t},x_{r},y_{l},n_{k}\right)=IFFT_{k_{i}}\left[sS\left(x_{t},x_{r},y_{l},k_{i}\right),MN_{f}\right]$$

The solution of $n_{k}$ is the same as that of (12) and ΔR is

(15) $$\Delta R=\frac{1}{MN_{f}-1}\cdot \frac{2\pi }{\Delta k}=\frac{N_{f}-1}{MN_{f}-1}\frac{2\pi }{B_{k}}$$

Generally, it is believed that the $E\left(x_{t},x_{r},y_{l},k_{i}\right)$ is negligible by satisfying $\left|R_{m}-R_{n}\right|\ll \lambda_{\min}$, that is $\Delta R\ll \lambda_{\min}$, where $\lambda_{\min}$ is the smallest wavelength within the frequency band. Therefore, the number of inverse FT sampling points should satisfy the following:

(16) $$MN_{k}\gg \frac{2\pi }{B_{k}\lambda_{\min}}\left(N_{f}-1\right)$$

As for a linearized inverse scattering problem, the target reflectivity function can be solved as follows:

(17) $$o\left(x,y,z\right)\approx ∭dx_{t}dx_{r}dy_{l}\cdot ss\left(x_{t},x_{r},y_{l},n_{k}\right)\exp\left(jk_{0}R_{M}\right)$$

To improve the precision of the reconstructed image, the phase error term $E\left(x_{t},x_{r},y_{l},k_{i}\right)$ can be compensated as follows:

(18) $$C=\exp\left[j\frac{N_{k}-1}{2}\Delta k\cdot \Delta R\right]$$

The final proposed algorithm after compensation can be expressed as follows:

(19) $$\begin{array}{l} I_{proposed}\left(x,y,z\right)\\ =∭dx_{t}dx_{r}dy_{l}\cdot {\left.ss\left(x_{t},x_{r},y_{l},n_{k}\right)\right|}_{n_{k}=\mathrm{int}\left(\frac{R_{M}}{\Delta R}\right)}\\ \cdot \exp\left(jk_{0}R_{M}\right)\cdot C \end{array}$$

### 2.3. The CF-Proposed Algorithm

CF is defined as the ratio of the coherent power $P_{coh}$ to the incoherent power $P_{inc}$ of the target reflectivity function [18], as follows:

(20) $$CF\left(x,y,z\right)=\frac{P_{coh}\left(x,y,z\right)}{P_{inc}\left(x,y,z\right)}$$

Combined with Formula (19), $P_{coh}$ and $P_{inc}$ in (20) can be defined as:

(21) $$P_{coh}\left(x,y,z\right)={\left|o\left(x,y,z\right)\right|}^{2}$$

(22) $$P_{inc}\left(x,y,z\right)={∭dx_{t}dx_{r}dy_{l}\cdot \left|{\left.ss\left(x_{t},x_{r},y_{l},n_{k}\right)\right|}_{n_{k}=\mathrm{int}\left(\frac{R_{M}}{\Delta R}\right)}\cdot \exp\left(jk_{0}R_{M}\right)\cdot C\right|}^{2}$$

Simply calculate the incoherence factor during image reconstruction to suppress the sidelobe. Then, the final reconstructed image can be expressed as:

(23) $$I_{CF-proposed}=I_{proposed}\cdot CF\left(x,y,z\right)$$

The proposed algorithm presents a 3D reconstruction scheme by performing IFFT on the wave number to speed up the traditional BP algorithm, then can be easily removed the coherent integrals of the k domain. The error compensation term is also analyzed to reduce the error. Based on the above formulation, the 3D reconstruction scheme can be separated into the following eight steps, as shown in Table 1:

### 2.4. Computation Complexity

The computational complexity of the proposed algorithm will be analyzed in this section. We use floating-point arithmetic to calculate the computational load required by the proposed algorithm. According to the above imaging formulas, the computation load of the proposed algorithm and traditional BPA can be obtained as follows:

(24) $$\begin{array}{ll} C_{proposed} & =8N_{xt}N_{xr}N_{yl}N_{x}N_{y}N_{z}\\ & +40N_{xt}N_{xr}N_{yl}MN_{f}\log_{2}\left(FN_{f}\right) \end{array}$$

(25) $$C_{BP}=8N_{xt}N_{xr}N_{yl}N_{f}N_{x}N_{y}N_{z}$$

(26) $$\begin{array}{ll} C_{CF-proposed} & =14N_{xt}N_{xr}N_{yl}N_{x}N_{y}N_{z}\\ & +40N_{xt}N_{xr}N_{yl}MN_{f}\log_{2}\left(FN_{f}\right) \end{array}$$

where $N_{xt}$, $N_{xr}$ denote the numbers of transmitters and receivers, $N_{yl}$ represents the number of scanning points in the y-direction. Let $N_{x}$, $N_{y}$, and $N_{z}$ denote the voxel in three dimensions, and $N_{x}$ is the number of the voxels in the x-orientation of the 3D focused image.

To further simplify the comparison, we assume that $N_{xt}=N_{xr}=\sqrt{N}$ and $N_{yl}=N_{x}=N_{y}=N_{z}=N_{k}=N$, then the computational complexity of (20) and (21) can be expressed more intuitive:

(27) $$C_{Proposed}=O\left(N^{5}\right)$$

(28) $$C_{BP}=O\left(N^{6}\right)$$

(29) $$C_{CF-Proposed}=O\left(N^{5}\right)$$

As shown in (22) and (23), the computational complexity of the proposed algorithm is much lower than that of the conventional BPA. According to (29), it can be seen that the CF-proposed algorithm has the same quantum of computational complexity.

### 2.5. Error Analysis

The main error is caused by neglecting the error term $E\left(x_{t},x_{r},y_{l},k_{i}\right)$ in Formula (9), and by the compensation operation in Formula (18), the residual maximum can be expressed as follows:

(30) $$\begin{array}{ll} PHE & ={\left|C\cdot E\left(x_{t},x_{r},y_{l},k_{i}\right)\right|}_{\max}\\ & =\frac{N_{f}-1}{2}\Delta k\cdot \Delta R\\ & \approx \frac{B_{k}}{2}\cdot \Delta R=\frac{N_{f}-1}{MN_{f}-1}\pi \approx \frac{1}{M}\pi \end{array}$$

Such a phase error can be controlled at a very low level. We can easily control the error to about 0.001π. To ensure imaging quality for each area, the maximum phase error should be lower than 0.25π. Thus the imaging error generated by the proposed algorithm is negligible. Additionally, considering the case of removing the compensation term. The maximum error between the proposed algorithm and the traditional BPA is about 2PHE, this will also not have a significant impact on the reconstruction results. Therefore, in practical applications, we can consider neglecting the effect brought by the error term.

## 3. Numerical Simulation and Experimental Verification

In this section, simulation experiments of point targets and letter A scattering models are carried out, and we also design a schematic prototype for experiments to verify the effectiveness of the algorithm. The advantages of the proposed algorithm over the conventional BP algorithm are demonstrated by the comparison of the computational speed, and the superiority of the proposed algorithm over the wave number domain algorithm is verified by setting up sparse array experiments. The specific parameter settings for simulation and experiments are shown in Table 2.

### 3.1. Simulation Results of Point Targets

**Figure 2:**
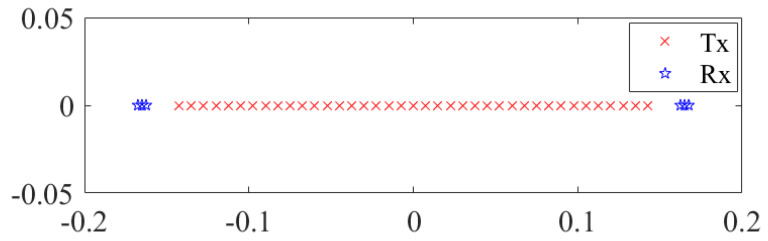
The MIMO-SAR array as applied in the simulation.

The 1D MIMO array with 39 transmitters and 6 receivers applied in this simulation is shown in Figure 2. Seven reference target points are set with the center point 0.7 m away from the array in the simulation scenario. All the reconstructed images are divided into 101 × 101 × 100 voxels, and the voxel size is 1 mm in the azimuth dimension. The echo signal of the scatterer is calculated by MATLAB, which uses echo signal model simulation.

**Figure 3:**
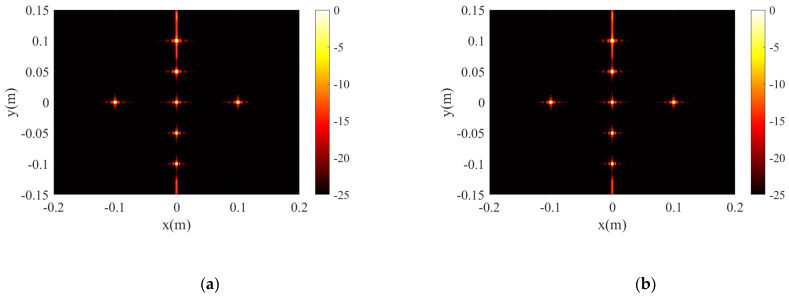

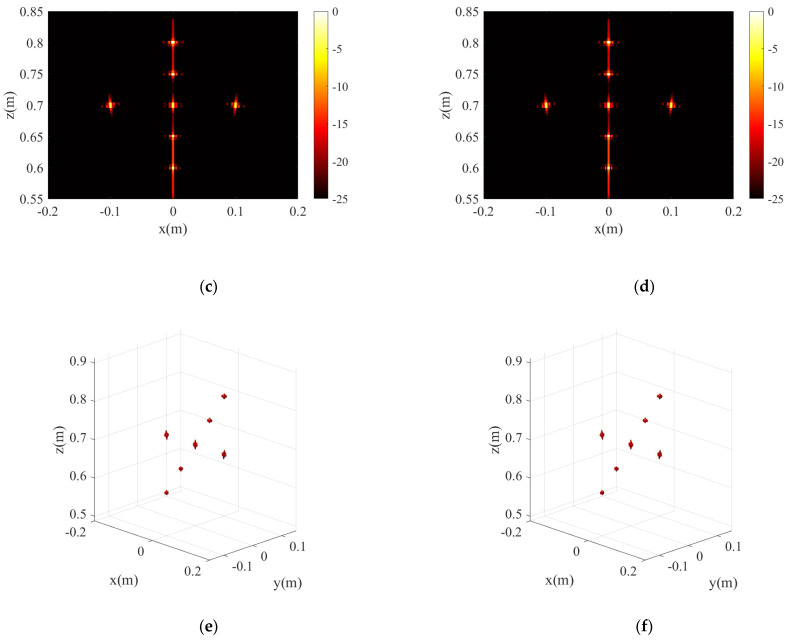
The simulation results for point targets. (**a**,**c**,**e**) are the 2D XY, 2D XZ, and 3D projection results of BPA. (**b**,**d**,**f**) are the 2D XY, 2D XZ, and 3D projection results of the proposed algorithm.

**Figure 4:**
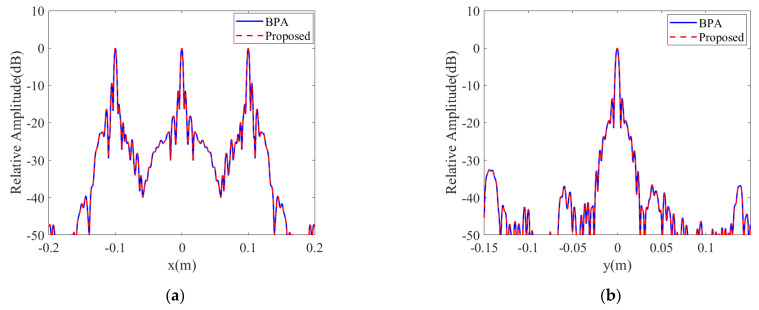
(**a**) The 1D profile along the *x*-axis of the center point. (**b**) The 1D profile along the *y*-axis of the center point.

Figure 3 gives the projection of BPA and the proposed algorithm in XY and XZ planes, respectively, and the 3D reconstruction results with dynamic range limited to −10 dB. The 1D profile along *x*-axis and *y*-axis of the center point is given in Figure 4. The simulation results verify that both the proposed algorithm and the BP algorithm perform excellent reconstruction of the point target.

### 3.2. Simulation Results of Letter A Scattering Models

**Figure 5:**
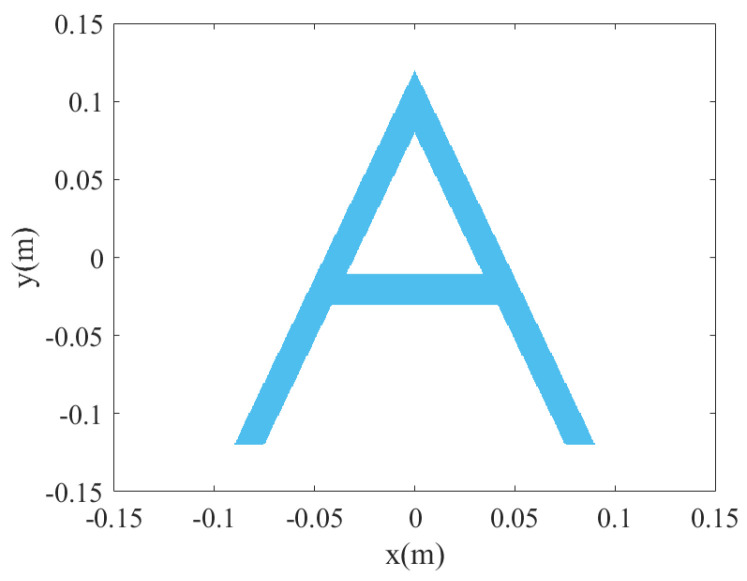
The ideal letter A scattering model.

**Figure 6:**
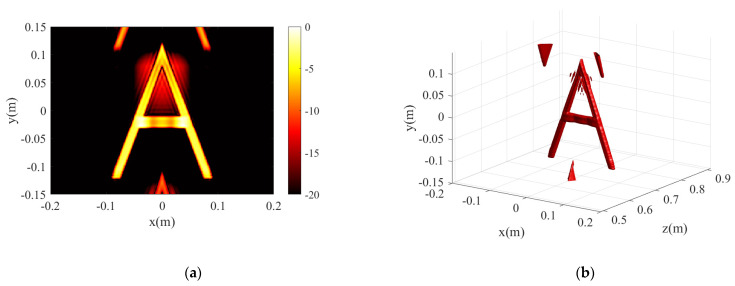

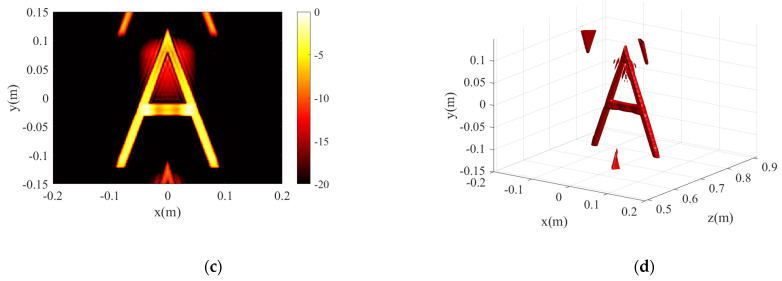
The simulation results for model A. (**a**,**b**) are the 2D and 3D projection results of BPA. (**c**,**d**) are the 2D and 3D projection results of the proposed algorithm.

To further evaluate the performance of the proposed algorithm, we set an ideal letter A scattering model for simulation. The echo signal of the MIMO-SAR is calculated using the following formula:

(31) $$sS\left(x_{t},x_{r},y_{l},k_{i}\right)=\sum_{i,j,m}o\left(x_{i},y_{j},z_{m}\right)\exp\left[-jk_{i}\left(R_{T}+R_{R}\right)\right]$$

where $o\left(x_{i},y_{j},z_{m}\right)$ denotes the reflectance function of the target cell $\left(x_{i},y_{j},z_{m}\right)$, which we set to 1. The model shown in Figure 5 is placed at a distance of 0.7 m from the array. The 3D and 2D simulation results are shown in Figure 6, where the 3D dynamic range is limited to −12 dB. Both algorithms achieve a well-reconstructed ideal letter A scattering model. For some artifacts on the periphery, it is a grating-lobes problem due to the sparsity in the y-scan direction, which is also caused by the close proximity of the target. Of course, these grating lobes can be removed by certain methods, the CF method in the proposed algorithm also has some suppression effect on the grating lobes, but this is not considered in this paper.

## 4. Lab Experiments Results

### 4.1. Lab Experiment 1

**Figure 7:**
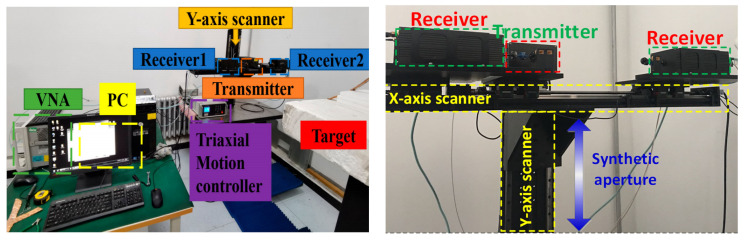
Laboratory experiment setup and photograph of the 3D scanner of the experiment setup.

The proposed algorithm is further verified experimentally by self-developed millimeter-wave MIMO scanning radar based on a microwave Vector Network Analyzer (VNA), which is shown in Figure 7, while the main parameters of the experiment are listed in Experiment 1 of Table 2.

Due to the width limitation of the antenna front-end, the interval between the adjacent transmitter and receiver in the experiment is 2 cm. The total length of the array is 0.335 m. The fourth motor is used to control the horizontal guide rail to scan in the vertical direction to achieve a synthetic aperture. After completing a round of horizontal transmission and reception, the horizontal guide railway moves 5 mm along the vertical y-direction, and the next round of data acquisition starts.

**Figure 8:**
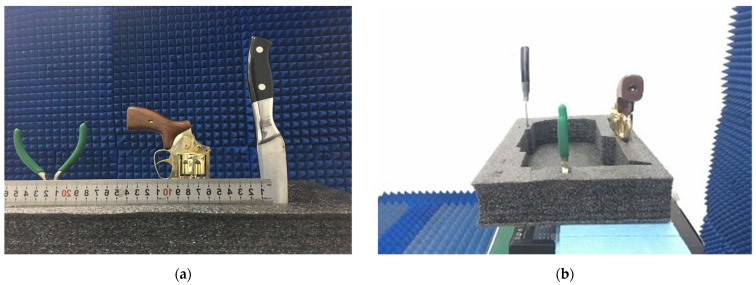
Photograph of three targets. (**a**) Photo of the front view of three targets. (**b**) Photo of the side view of three targets.

**Figure 9:**
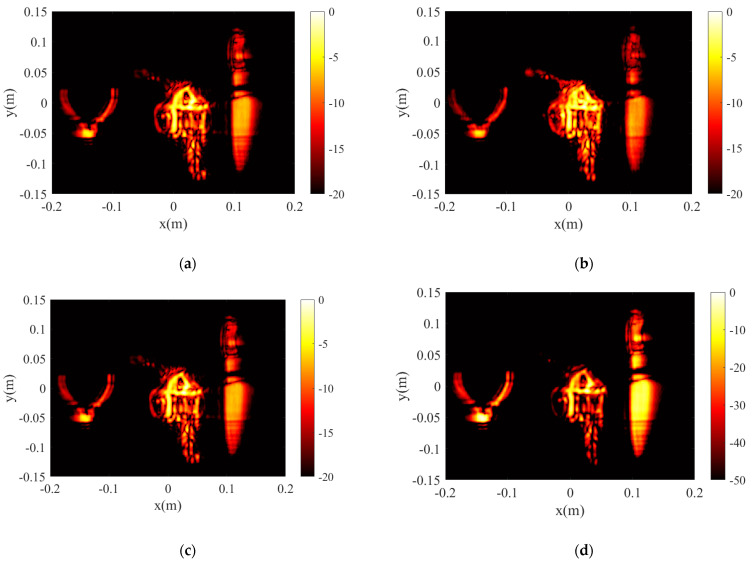
Experiment one’s results. (**a**) Result of BPA. (**b**) Result of PSM. (**c**) Result of the proposed algorithm. (**d**) Result of the CF-proposed algorithm.

The reconstructions for the three models in Figure 8 are compared in Figure 9a–d. The reconstruction results of all the targets are shown in the dynamic range of −20 dB, which is mainly determined by the number of array transceiver units, location, and signal-to-noise ratio of the measurement.

### 4.2. Lab Sparse Experiment 2

**Figure 10:**
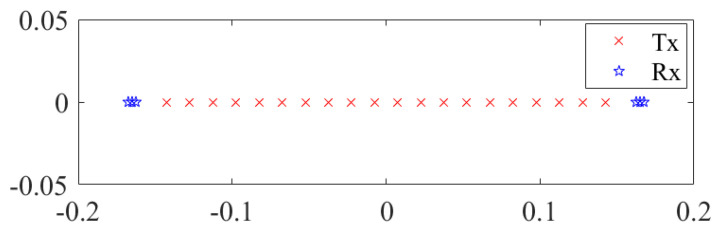
The MIMO-SAR sparse array applied in the simulation.

**Figure 11:**
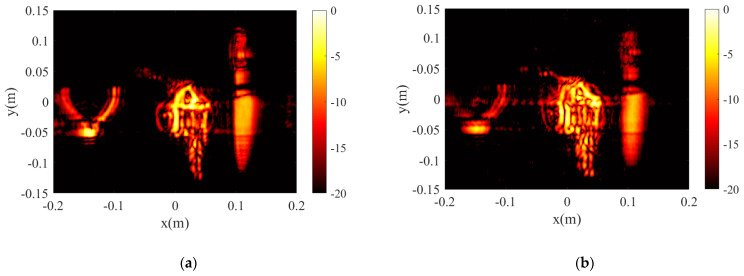

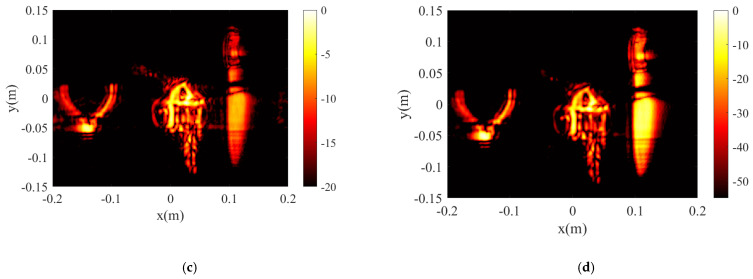
Sparse reconstructed image Experiment two’s results. (**a**) The result of the BPA after sparse. (**b**) The result of the PSM after sparse. (**c**) The result of the proposed algorithm after sparse. (**d**) The result of the CF-proposed algorithm after sparse.

To verify the advantages of the proposed algorithm over traditional wavenumber domain algorithms, comparative tests are conducted with the three targets in Figure 8. PSM [17], as a classical wavenumber domain algorithm, is used in this paper to compare with the proposed algorithm. The number of transmitting array elements is reduced from 39 to 20 by equally spaced sparsity, while the number of receiving array elements remains unchanged, as shown in Figure 10. The parameters of the experiment are consistent with Experiment 2 in Table 2. Comparative test results are shown in Figure 11. For uniformly sparse arrays, the proposed algorithm still exhibits good image reconstruction performance, as shown in Figure 11c; compared to Figure 9c, the side lobe of the reconstructed target image has significant side lobes, which can be easily removed by certain measures. For the PSM algorithm, the pre-sparing reconstructed target image is shown in Figure 9b, while the reconstructed target image after sparse is shown in Figure 11b. The details of the target have been severely distorted after sparsity, leading to a serious loss of detail in the reconstructed image. This is also the biggest limitation of wavenumber domain algorithms. Comparative tests indicate that the proposed algorithm has better adaptability to array element sparsity than the wavenumber domain algorithm. This can reduce the hardware cost of the system to a certain extent. Figure 11d shows the 3D reconstruction results of the CF-proposed. Compared with Figure 11c, the sidelobe suppression effect is obvious.

## 5. Discussion

To compare the performance of the algorithms more clearly, the structural similarity (SSIM) evaluation index is introduced to evaluate the focusing performance of 2D images [20,21,22]. The calculation of SSIM can be expressed by the following formula:

(32) $$SSIM\left(I_{1},I_{2}\right)=\frac{\left(2\mu_{1}\mu_{2}+C_{1}\right)\left(2\sigma_{12}+C_{1}\right)}{\left(\mu_{1}^{2}+\mu_{2}^{2}+C_{1}\right)\left(\sigma_{1}^{2}+\sigma_{2}^{2}+C_{1}\right)}$$

where $I_{1}$ represents the reference image and $I_{2}$ represents the image to be evaluated. $\mu_{1}$, $\mu_{2}$, $\sigma_{1}$, $\sigma_{2}$ and $\sigma_{12}$ are the average, variance, and cross-covariance of the two images, respectively. $C_{1}$ and $C_{2}$ are constants that maintain stability in order to keep the denominator from being 0, usually take $C_{1}={\left(K_{1}L\right)}^{2}$, SSIM is calculated for values from 0 to 1, and the larger its value, the smaller the gap between the image to be evaluated and the reference image.

Using the BPA as a reference, the difference between the proposed algorithm, PSM, and BPA are compared by calculating SSIM. Then, we evaluate the effectiveness of the algorithm using computational time and SSIM for the simulations and experiments in Section 3 and Section 4; as shown in Table 3, it can be seen that the proposed algorithm has obvious efficiency advantages. Table 3 also shows the computation time of the CF-proposed algorithm in two sets of experiments to highlight the impact of CF on the computational efficiency of the proposed algorithm.

## 6. Conclusions

In this paper, a modified BPA is proposed for 3D reconstruction based on MIMO-SAR. To address the problem of the high redundancy of the traditional BPA and its iteration operations, the wavenumber dimension data are improved, and the inverse FT method is adopted to accelerate the algorithm without damaging the image quality. The Experiment 1 results in Table 3 show that, compared with BPA, the computational efficiency of the proposed algorithm is significantly improved, with an SSIM value of 0.9960 and SSIM value of 0.8570 for PSM, indicating that the proposed algorithm performs 3D reconstruction without compromising the imaging quality. The results of Experiment 2 show that the effect of array sparsity likewise does not degrade the quality of the reconstructed images by the proposed algorithm, while the quality of the images reconstructed by PSM is severely degraded. Experimentally, it is proved that although the wave number domain algorithm has obvious advantages in reconstruction speed, it loses imaging accuracy to a certain extent. In an overly sparse array, the wavenumber domain algorithm reconstruction effect is severely degraded, and the original information of the target cannot be recovered; the proposed algorithm does not have this problem with BPA.

In addition, the CF-proposed algorithm is used to reconstruct the images under each of the two sets of experiments to achieve the suppression of the sidelobe energy without losing the image quality. The main contribution of this paper is as follows: The first is to propose an algorithm that has the same reconstructed image quality as the traditional BPA but is much more efficient than the BPA. The second is to combine CF with the proposed algorithm to suppress sidelobe energy. 

## Figures and Tables

**Table 1:** Detailed procedures of the CF-proposed algorithm for MIMO-SAR imaging.

Input: Recorded the Original Echo for Each Channel.
Step 1. Obtain 4D echo data $sS\left(x_{t},x_{r},y_{l},k_{i}\right)$ based on the MIMO-SAR system. Step 2. Calculate the condition that the inverse FT sampling points $MN_{f}$ meet according to Formula (16). Step 3. Perform an inverse $MN_{f}$ FT on the wavenumber dimension of the echo signal in Formula (1). Step 4. Calculate compensation item (14). Step 5. Calculate the incoherent power $P_{inc}$. Step 6. Perform coherent accumulation in three dimensions $x_{t}$, $x_{r}$, $y_{l}$. Step 7. Calculate the coherent factor $CF\left(x,y,z\right)$ according to (20). Step 8. Reconstruct the target according to (15) and (23).
Output: 3D MIMO-SAR imaging results.

**Table 2:** Parameters of simulation and experiments.

Parameter (Unit)	Simulation	Experiment 1	Experiment 2
Frequency (GHz)/interval (MHz)	75 to 110/350	75 to 110/175	75 to 110/175
Number of Tx/interval (mm)	39/7.5	39/7.5	20/15
Number of Rx/interval (mm)	6/2.5	6/2.5	6/2.5
Scan points/length (mm)	60/5	60/5	60/5
Imaging range (m × m × m)	0.4 × 0.3 × 0.4	0.4 × 0.3 × 0.85	0.4 × 0.3 × 0.85

**Table 3:** Processing times and SSIM of different algorithms.

	Algorithms	Processing Times (s)	SSIM
Simulation	BP	7213.29	1
	Proposed algorithm	167.35	0.9970
Experiment 1	BP	34021.66	1
	PSM	382.56	0.8507
	proposed algorithm	463.34	0.9960
	CF-proposed	501.49	/
Experiment 2	BP	17114.23	1
	PSM	188.51	0.7762
	proposed algorithm	232.70	0.9956
	CF-proposed	277.90	**/**

## Data Availability

No new data were created or analyzed in this study. Data sharing is not applicable to this article.
